# Zinc Transporter Gene Variants (SLC30A and SLC39A) in Human Disease: From Metal Homeostasis Disruption to Convergent Signaling Pathways

**DOI:** 10.1155/humu/8829745

**Published:** 2026-09-29

**Authors:** Bo Sun, Jing Ma, Xiaohong Bai, Shusong Wang

**Affiliations:** ^1^ Department of Gynecology and Obstetrics, Tianjin Medical University General Hospital, Tianjin, China, tjmugh.com.cn; ^2^ Tianjin Key Laboratory of Female Reproductive Health and Eugenics, Tianjin Medical University General Hospital, Tianjin, China, tjmugh.com.cn; ^3^ Hebei Key Laboratory of Reproductive Medicine, Hebei Reproductive Health Hospital, Shijiazhuang, Hebei, China

**Keywords:** gene mutation, single nucleotide polymorphisms, zinc, zinc transporter protein

## Abstract

Zinc, an indispensable trace element, performs critical functions in human physiology. As a versatile cofactor, zinc participates in over 1000 enzymatic reactions and regulates more than 2000 transcription factors, orchestrating diverse cellular signaling pathways. The transmembrane transport of zinc is mediated by specialized transporter proteins, which are categorized into two families: zinc transporters (ZnTs) and Zrt‐ and Irt‐like proteins (ZIPs). These transporters are pivotal for maintaining cellular and systemic zinc homeostasis. Pathogenic variants in ZnT/ZIP genes are associated with a spectrum of severe human diseases. This review systematically examines the pathophysiological connections between ZnT/ZIP gene variants and human diseases, providing a conceptual framework for future mechanistic investigations.

## 1. Introduction

Zinc is an essential trace element that plays a critical role in humans, ranking as the second most abundant trace element after iron. A healthy adult body contains approximately 2~3 g of zinc, with 60% located in skeletal muscle, 30% in bone, 5% in the liver and skin, and the remaining 2%~3% distributed in other tissues [1]. Zinc is vital for numerous physiological processes, including RNA synthesis, DNA synthesis and damage repair, protein synthesis, cell proliferation and differentiation, antioxidant defense, immune protection, and tissue repair [2, 3]. Zinc serves as a cofactor for over 1000 enzymatic reactions and more than 2000 transcription factors, playing a role in nearly every aspect of cellular signaling. Consequently, zinc has long been a focus of scientific interest. The human body lacks a specialized zinc storage system, necessitating daily zinc intake from food to compensate for losses. Due to the hydrophobic nature of zinc ions, they cannot readily cross the plasma membrane by simple diffusion. Instead, zinc ion transport across membranes requires the assistance of zinc transporter (ZnT) proteins.

ZnT proteins are categorized into two families: ZnTs and Zrt‐ and Irt‐like proteins (ZIPs) [4]. The ZnT family, encoded by the solute carrier family 30 member A (*SLC30A*) gene family, has 10 members in the human body and is mainly responsible for transferring zinc out of the cytoplasm to modulate intracellular zinc concentrations [4]. Conversely, the ZIP family, encoded by the solute carrier family 39 (SLC39A) gene family, comprises 14 members and is primarily responsible for transporting zinc into the cytoplasm to elevate cytosolic zinc levels [4]. These transporters exhibit distinct subcellular localizations, enabling compartment‐specific zinc regulation critical for their biological functions. The localization of ZnT proteins in cells is shown in Figure 1. In humans, ZnT and ZIP family members display tissue‐specific expression patterns that collectively maintain systemic zinc homeostasis. Detailed information on the tissue distribution and functions of the ZnT and ZIP families is shown in Tables 1 and 2.

**Figure 1:**
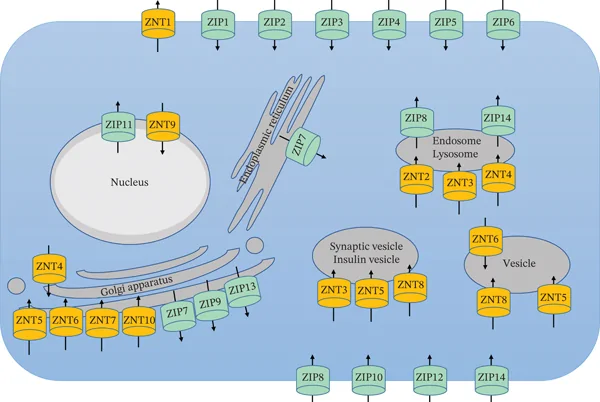
Localization of zinc transporter proteins in cells. Arrows represent the direction of movement of zinc ions.

**Table 1:** Localization and physiological function of ZnTs.

ZnT (*SLC30*)	Subfamily	Localization	Physiological function	References
ZnT1 (*SLC30A1*)	II	Kidney, placenta, intestines, and erythrocytes, localized in the cell membrane and cytoplasm	Blood zinc transport, intestinal zinc absorption, renal reabsorption, and secretion	[4, 5]
ZnT2 (*SLC30A2*)	III	Prostate, breast, intestines, pancreas, and kidney, localized in the lysosomes and endosomes	Regulation of glandular secretion	[6]
ZnT3 (*SLC30A3*)	III	Hippocampus and cerebral cortex, localized in the endosomes and synaptic vesicles	Neuronal cellular zinc homeostasis	[4, 7]
ZnT4 (*SLC30A4*)	III	Cerebral cortex, mammary gland, small intestine, and epithelial cells, localized in the Golgi apparatus, lysosomes, and endosomes	Zinc secretion in milk and intestinal zinc absorption	[4, 8, 9]
ZnT5 (*SLC30A5*)	II	Expressed in various tissues and organs, localized in the Golgi apparatus and vesicles	Osteoblast maturation and cardiovascular development	[4, 10]
ZnT6 (*SLC30A6*)	III	Liver, kidneys, small intestine, and brain, localized in the Golgi apparatus and vesicles	Translocation of zinc to the Golgi apparatus	[11]
ZnT7 (*SLC30A7*)	II	In mice, ZnT7 is expressed in the small intestine, lung, liver, kidney, spleen, heart, and brain, localized in the Golgi apparatus	Transportation of zinc to the Golgi apparatus	[1, 4]
ZnT8 (*SLC30A8*)	III	Pancreatic *β*‐cells, localized in the vesicle membrane of insulin secretion granules	Insulin storage and secretion	[1, 4, 12]
ZnT9 (*SLC30A9*)	II	Cerebellum, skeletal muscle, thymus, and kidneys, localized in the cell nucleus	Involved in cellular zinc ion transport	[13, 14]
ZnT10 (*SLC30A10*)	I	Brain, liver, and retina, localized in the Golgi apparatus	Zinc ion and manganese ion transport	[15]

**Table 2:** Localization and physiological function of ZIPs.

ZIP (*SLC39A*)	Localization	Physiological function	References
ZIP1 (*SLC39A1*)	Expressed in various tissues and organs, localized in the cell membrane and cytoplasm	Embryonic development	[16–18]
ZIP2 (*SLC39A2*)	Liver, skin, and ovary, localized in the cell membrane	Embryonic development and cellular uptake of zinc	[19–21]
ZIP3 (*SLC39A3*)	Expressed in various tissues and organs, localized in the cell membrane	Embryonic development, T‐cell development, and reabsorption of zinc during mammary cell formation	[22–25]
ZIP4 (*SLC39A4*)	Intestine, skin, brain, and blood vessels, localized in the cell membrane	Intestinal zinc absorption	[26, 27]
ZIP5 (*SLC39A5*)	Eyes, intestines, kidneys, liver, and pancreas, localized in the cell membrane	Pancreatic zinc accumulation, intestinal zinc excretion, and insulin secretion	[28–34]
ZIP6 (*SLC39A6*)	Expressed in various tissues and organs, localized in the cell membrane	The aggressiveness of malignant tumors	[35, 36]
ZIP7 (*SLC39A7*)	Expressed in various tissues and organs, localized in the Golgi apparatus and endoplasmic reticulum	The maintenance of normal immune function	[37, 38]
ZIP8 (*SLC39A8*)	Liver, kidneys, lungs, testicles, and brain, localized in the cell membrane	Immune function, heart development, bone metabolism, and manganese metabolism	[39–41]
ZIP9 (*SLC39A9*)	Skin and testis, localized in the Golgi apparatus	Androgen receptor and zinc ion transport	[42–44]
ZIP10 (*SLC39A10*)	Expressed in various tissues and organs, highly expressed in the skin, brain, liver, and small intestine, localized in the cell membrane	Embryonic development and epidermal and hair follicle development	[45–48]
ZIP13 (*SLC39A13*)	Bone, tooth, and hard and connective tissues, localized in the Golgi apparatus	Growth and development and transportation of zinc ions from the Golgi to the cytoplasm	[49–51]
ZIP14 (*SLC39A14*)	Heart, liver, intestines, pancreas, and heart, localized in the cell membrane and endosomes	Growth and development and the function of transporting metal ions	[52–55]

As key regulators of zinc homeostasis, ZnTs and ZIPs play indispensable roles in human physiology. Pathogenic mutations in ZnT/ZIP genes have been implicated in various disorders, including transient neonatal zinc deficiency (TNZD), acrodermatitis enteropathica (AE), and spondylocheirodysplastic Ehlers–Danlos syndrome (SCD‐EDS) [56–58]. A single nucleotide polymorphism (SNP) is a variation in DNA caused by a change in a single nucleotide base. As the predominant form of genetic variation, SNPs have garnered significant research attention in contemporary genomics. Emerging evidence demonstrates that functional polymorphisms in ZnT/ZIP genes exhibit robust associations with diverse disease phenotypes. *SLC30A3* rs6547521 shows a sex‐specific association with schizophrenia risk in females [59]. *SLC30A5* mutations are associated with perinatal cardiomyopathy manifesting as fetal mortality [60]. *SLC30A7* is a candidate pathogenic gene for a rare genetic disease characterized by congenital brain malformations and neurological disorders [61]. *SLC30A8* rs13266634 C allele confers increased susceptibility to Type 2 diabetes mellitus (T2DM) [62]. Mutations in the *SLC30A10* gene lead to dystonia and hypermanganism [63]. *SLC39A11* variants demonstrate oncogenic potential in renal and bladder carcinogenesis [64]. The advent of genome‐wide association studies (GWAS) has significantly advanced our understanding of genotype–phenotype correlations between ZnT variants and disease pathogenesis. By systematically screening millions of SNPs across the genome, GWAS enables the identification of disease‐associated genetic markers with unprecedented resolution. Collectively, this review systematically evaluates the pathophysiological connections between ZnT/ZIP genetic variations and human disorders, providing a conceptual framework for future mechanistic investigations.

## 2. ZnTs (SLC30A)

### 2.1. ZnT1 (*SLC30A1*)

ZnT1 demonstrates ubiquitous expression in vivo, with predominant plasma membrane localization (Table 1) [5]. In intestinal epithelium, ZnT1 is strategically positioned at the basolateral membrane, mediating zinc efflux into the portal circulation [65]. Functionally, ZnT1 plays a critical role in murine embryogenesis by facilitating maternal‐to‐embryonic zinc transfer, as evidenced by embryonic lethality in *SLC30A1* homozygous knockout models [66]. In human pathophysiology, Rege et al. identified somatic *SLC30A1* mutations (p.L51_A57del, *n* = 3; p.L49_L55del, *n* = 2) in primary aldosteronism patients [67]. SLC30A1 mutations trigger abnormal sodium influx, leading to membrane depolarization, which in turn promotes calcium influx, ultimately upregulating aldosterone synthase (CYP11B2) expression and resulting in aldosterone excess and hypertension [67]. This signaling cascade ultimately stimulates aldosterone synthase expression and enhances aldosterone biosynthesis. Sadler et al. identified, through a large‐scale GWAS, that SLC30A1 genetic variants are associated with cardiometabolic diseases, including hypertension, dyslipidemia, and coronary heart disease. This represents the first demonstration of an association between SLC30A1 genetic variants and cardiometabolic diseases at the population genetics level [68].

### 2.2. ZnT2 (*SLC30A2*)

ZnT2 exhibits tissue‐specific expression in the prostate, mammary gland, intestine, pancreas, and kidney, with primary localization to lysosomal and endosomal compartments (Table 1). In mammary epithelium, ZnT2 plays a pivotal role in mediating zinc secretion into breast milk. Pathogenic variants in *SLC30A2* impair zinc secretion, resulting in decreased milk zinc concentration [6]. Under physiological conditions, breast milk zinc adequately supports infant growth and development. Insufficient milk zinc induces TNZD in exclusively breastfed infants, which remains refractory to maternal zinc supplementation [56]. Direct zinc supplementation to infants effectively ameliorates TNZD symptoms. Notably, mothers remain asymptomatic despite their infants′ zinc deficiency [56]. This observation indicates that TNZD stems from impaired zinc secretion rather than maternal zinc deficiency. The *SLC30A2* mutations represent a primary genetic etiology of TNZD. Multiple studies confirm that maternal *SLC30A2* mutations are causally linked to infant TNZD [6, 69]. Seo and Kelleher demonstrated that *SLC30A2* variants (rs35235055 and rs35623192) compromise mammary zinc secretion [69]. The rs35235055 C allele induces lysosomal zinc accumulation, thereby reducing milk zinc secretion. The rs35623192 T allele results in ZnT2 mislocalization to the Golgi apparatus [69]. This aberrant localization disrupts cellular zinc homeostasis without altering Golgi zinc content, ultimately impairing extracellular zinc secretion. Itsumura et al. identified additional *SLC30A2* variants (rs148861822, rs200520278, rs201084300, and rs377192955) associated with impaired mammary zinc secretion [70]. Collectively, these findings establish maternal *SLC30A2* mutations as major contributors to TNZD through impaired mammary zinc transport. Carter et al. systematically summarized all currently known pathogenic SLC30A2 variants responsible for TNZD [71]. Although the population prevalence of pathogenic SLC30A2 variants remains uncertain, incorporating SLC30A2 into maternal carrier screening panels would enable the proactive identification of women harboring known pathogenic SLC30A2 variants, thereby facilitating timely neonatal zinc supplementation and reducing the incidence of TNZD [71].

### 2.3. ZnT3 (*SLC30A3*)

ZnT3 exhibits region‐specific expression in the brain, with particularly high abundance in the hippocampus and cerebral cortex (Table 1) [7]. This distribution pattern implicates ZnT3 in modulating central nervous system functions, and its dysregulation has been associated with various neurological disorders. Genetic polymorphisms in *SLC30A3* have been linked to increased schizophrenia susceptibility. ZnT3 was found to physically interact with the synaptic scaffold protein Shank3 in mice [72]. The trans‐synaptic ZnT3–Shank3 coupling is essential for maintaining dendritic spine structure and function [72]. Shank3 is a human population risk gene associated with autism spectrum disorder [72]. Loss of ZnT3 function leads to reduced dendritic spine density and impaired synaptic transmission [72]. ZnT3 knockout mice exhibit behavioral deficits relevant to both autism spectrum disorder and schizophrenia [72]. In a UK cohort study, Perez‐Becerril et al. identified four *SLC30A3* SNPs (rs6547521, rs11126936, rs2083363, and rs11126931) showing significant association with schizophrenia risk [59]. Notably, this association exhibited sexual dimorphism, being restricted to female patients [59]. These findings indicate a female‐specific genetic predisposition mediated by *SLC30A3* variants. Subsequent replication studies in larger European cohorts confirmed this gender‐specific association, further validating the *SLC30A3*‐schizophrenia link in females [73]. This difference may be related to the distinct effects of sex hormones, such as estrogen and testosterone, on the expression or function of ZnT3. Estrogen can regulate the expression and activity of ZnTs, and the levels of estrogen in females may make them more sensitive to changes in ZnT3 function [74].

### 2.4. ZnT4 (*SLC30A4*)

ZnT4 demonstrates broad tissue distribution, with particularly high expression in mammary and intestinal epithelial cells (Table 1). Functionally, ZnT4 primarily mediates zinc secretion into milk and facilitates intestinal zinc absorption. A previous study found that genetic ablation of *SLC30A4* in mice results in impaired mammary gland function, manifesting as secretory defects and premature involution. Phenotypic analysis revealed severe mammary gland abnormalities, including a 25% reduction in gland weight, a 50% decrease in milk production, a 50% lower lactose content, and a 35%–50% reduction in milk zinc concentration. This nutritional deficiency leads to neonatal mortality during lactation [8]. This phenotype, known as the lethal milk (lm) mutation, represents a well‐characterized model of zinc transport deficiency. Despite its critical role in murine zinc transport, *SLC30A4* mutations have not been implicated in human lactation disorders or TNZD [9]. In the intestinal epithelium, ZnT4 contributes to zinc homeostasis through its involvement in enterocyte zinc absorption. Notably, *SLC30A4* variants have not been associated with AE in human populations [75]. Beyond its epithelial functions, ZnT4 is expressed in testicular tissue, where it modulates testosterone biosynthesis via autophagy regulation through the PI3K/Akt/mTOR pathway, as evidenced by reduced androgen production following ZnT4 knockdown in Leydig cells [76].

### 2.5. ZnT5 (*SLC30A5*)

ZnT5 exhibits ubiquitous expression in human tissues and is crucial for maintaining zinc homeostasis in cardiac, neuromuscular, and skeletal systems (Table 1). Murine models with *SLC30A5* mutations display growth retardation, osteoporotic phenotypes, and cardiac conduction abnormalities [10]. In humans, *SLC30A5* mutations manifest more severe pathological consequences. Lieberwirth et al. identified biallelic loss‐of‐function variants in *SLC30A5* as causative factors for perinatal lethal cardiomyopathy [60]. Among four fetal cases, two pathogenic variants (c.832_836del and c.1981_1982del) were characterized [60]. These nonsense mutations result in complete functional ablation of ZnT5, ultimately causing perinatal cardiomyopathy and fetal demise [60]. Unlike the cases previously documented by Lieberwirth et al., which exhibited hydrops fetalis, cystic hygroma, and cardiac abnormalities, Dolgin et al. reported a case of severe neonatal hypotonia caused by a loss‐of‐function variant in SLC30A5 that impairs ZnT5 ZnT function [77]. This disruption leads to zinc metabolism dysregulation, resulting in clinical manifestations such as neuromuscular dysfunction, developmental delays, and feeding difficulties. The findings underscore the critical role of ZnT5 in zinc homeostasis and neuromuscular development.

### 2.6. ZnT6 (*SLC30A6*)

ZnT6 primarily localizes to the Golgi apparatus and vesicular compartments in various tissues, including the liver, kidney, and small intestine (Table 1) [11]. This transporter mediates zinc transport from the cytosol into Golgi/vesicular compartments and regulates zinc efflux pathways [11]. This transporter transports zinc from the cytosol into Golgi/vesicular compartments and regulates zinc efflux pathways [11]. Rheumatoid arthritis (RA), a prevalent chronic autoimmune disorder, is characterized by persistent inflammation causing progressive tissue destruction and functional impairment, with significant genetic contributions [78]. Zinc acts as an anti‐inflammatory factor, and low serum zinc levels can increase systemic inflammation and proinflammatory cytokines, thereby exacerbating the activity of RA [79]. Adelino et al. used the Health Assessment Questionnaire (HAQ) and the Clinical Disease Activity Index (CDAI) to assess the severity of RA in patients [80]. They found that RA patients carrying the *SLC30A6* rs430759 A/A genotype had significantly lower CDAI scores, indicating that the A/A genotype is associated with lower disease severity [80]. The rs430759 A>G polymorphism is located in the 3 ^′^ untranslated region (UTR) of the *SLC30A6* gene, and the presence of the A allele abolishes the binding site for miR‐4760‐3p, thereby upregulating the expression of *SLC30A6* [80]. This upregulation may modulate zinc levels and downregulate NF‐*κ*B activation, leading to reduced production of proinflammatory cytokines and ultimately lowering RA disease activity [80].

### 2.7. ZnT7 (*SLC30A7*)

ZnT7 demonstrates broad tissue distribution in humans, with significant expression in the pancreas, brain, and testicular tissues (Table 1). Experimental evidence indicates that ZnT7 modulates testosterone biosynthesis through the regulation of steroidogenic enzyme expression in Leydig cells, with ZnT7 knockdown significantly reducing androgen production [81]. In murine models, ZnT7 shows particularly high expression in the cerebellar cortex, suggesting its critical role in maintaining cerebellar zinc homeostasis. Huang et al. identified the first pathogenic *SLC30A7* mutations associated with severe clinical manifestations [82]. Specifically, they characterized *SLC30A7* mutations in siblings presenting with growth retardation, testicular hypoplasia, and bone marrow failure [82]. Genetic analysis revealed compound heterozygous mutations (NM_133496.5:c .21dup, p.Asp8ArgfsTer3, and c.842+15T>C) in the affected individuals [82]. This landmark discovery not only establishes *SLC30A7* as a disease‐associated gene but also demonstrates the therapeutic potential of hormone replacement therapy, providing new insights into the physiological roles of zinc and ZnT7 in human development [82]. Penon‐Portmann et al. proposed *SLC30A7* as a potential causative gene for Joubert syndrome (JS) [61]. JS, a neurodevelopmental disorder affecting cerebellar and brainstem formation, represents a classical ciliopathy [61]. Diagnostic hallmarks include the pathognomonic molar tooth sign on MRI, ataxia, and characteristic neurodevelopmental abnormalities. Genetic screening identified two *SLC30A7* variants (NM_133496.5:c.4T>C, p.Val136Ala and c.490_491delinsAG, p.His164Ser) in JS patients, further supporting its candidate gene status [61]. Functional characterization of these ZnT7 mutants may provide mechanistic insights into JS pathogenesis and inform therapeutic strategies [61].

### 2.8. ZnT8 (*SLC30A8*)

ZnT8 is predominantly expressed in pancreatic *β*‐cells, where it facilitates zinc transport into insulin secretory granules (Table 1). Within these vesicles, Zn^2+^ coordinates with insulin to form stable hexameric complexes, enabling proper insulin storage [83]. Pathogenic mutations in *SLC30A8* compromise zinc transport capacity, leading to reduced intragranular zinc levels, disrupted insulin storage/secretion, and elevated T2DM risk. T2DM prevalence has escalated dramatically globally, currently affecting over 170 million individuals. While multiple risk factors contribute to T2DM pathogenesis, genetic predisposition plays a pivotal role [12]. The seminal 2007 GWAS by Sladek et al. in a French cohort identified *SLC30A8* as a novel T2DM susceptibility locus, with the rs13266634 C allele conferring increased disease risk [62]. Specifically, rs13266634 C allele carriers exhibit a 14% higher T2DM risk compared to noncarriers [84]. Subsequent multiethnic studies across Asian, American, European, and Arab populations have consistently replicated this association, accounting for population stratification and environmental confounders [12, 85–88].

ZnT8 is also implicated in Type 1 diabetes mellitus (T1DM) pathogenesis, a condition marked by hyperglycemia resulting from autoimmune‐mediated *β*‐cell destruction and consequent insulin deficiency [89]. While T1DM pathogenesis remains incompletely understood, both autoimmune mechanisms and genetic predisposition are established risk factors [89]. ZnT8 represents a novel *β*‐cell‐specific autoantigen, with ZnT8 autoantibodies (ZnT8As) serving as valuable diagnostic biomarkers for T1DM [89]. ZnT8As are detectable during T1DM′s prodromal phase, facilitating early diagnosis [89]. ZnT8As are present in 60%–80% of early T1DM cases, and their combined detection with other autoantibodies enhances diagnostic sensitivity to 98% [90]. Furthermore, the *SLC30A8* rs13266634 polymorphism influences ZnT8A specificity, necessitating genotype‐specific interpretation of ZnT8A results to account for individual variation. Additionally, *SLC30A8* confers T1DM susceptibility, with rs2466293 showing significant association with disease risk [91].

Beyond T1DM, rs13266634 modulates gestational diabetes mellitus (GDM) risk and influences postrenal transplant outcomes. Benny et al. demonstrated that rs13266634 elevates GDM risk across diverse ethnic populations [92]. This polymorphism has emerged as a potential GDM biomarker [93]. Post‐transplantation diabetes mellitus (PTDM) represents a frequent complication following renal transplantation [94]. *SLC30A8* rs13266634 contributes to PTDM development and predicts poorer post‐transplant outcomes [94]. *SLC30A8* has emerged as a promising therapeutic target, with its functional characterization potentially informing novel diabetes treatment strategies.

### 2.9. ZnT9 (*SLC30A9*)

ZnT9 exhibits tissue‐specific expression in humans, with particularly high levels in the brain, kidney, and skeletal muscle (Table 1). It is crucial for maintaining cerebral zinc homeostasis, ensuring proper neurological function [13]. Disruption of brain zinc homeostasis may lead to diverse neurological pathologies. Pathogenic variants in SLC30A9 have been associated with Birk–Landau–Perez syndrome. Birk–Landau–Perez syndrome is a rare autosomal recessive cerebrorenal syndrome first described by Perez et al. Perez et al. identified that *SLC30A9* mutations disrupt intracellular zinc regulation, causing a previously unrecognized cerebrorenal syndrome [14]. This novel autosomal recessive disorder is characterized by early‐onset neurodegeneration, limb ataxia, muscle weakness, and progressive renal dysfunction [14]. Wagner et al. reported a novel case of an SLC30A9 mutation in a patient presenting with atypical chorioretinal degeneration, suggesting that SLC30A9‐associated disease may involve the retina [95]. This finding expands the phenotypic spectrum of SLC30A9‐related disorders and underscores their multisystem involvement [95].

### 2.10. ZnT10 (*SLC30A10*)

Initially characterized as a zinc‐specific transporter, ZnT10 was initially believed to mediate Zn^2+^ efflux from the cytoplasm to extracellular/organellar compartments to maintain cytosolic zinc homeostasis. Emerging evidence reveals that ZnT10 possesses dual ion transport capacity, specifically functioning as a primary manganese efflux transporter [15]. This dual functionality establishes ZnT10 as a critical regulator of systemic manganese homeostasis [96]. Pathogenic variants in *SLC30A10* are causally linked to hereditary hypermanganesemia [97–99]. Clinical studies demonstrate that *SLC30A10* mutations result in systemic manganese accumulation manifested as elevated blood manganese levels. Pathognomonic manganese deposition occurs predominantly in the basal ganglia, evidenced by characteristic T1‐weighted hyperintensity on brain MRI. Multisystem involvement includes the classic triad: hypermanganesemia, polycythemia, and hepatic cirrhosis, accompanied by neurological manifestations (dystonia and seizures) [63, 97, 100–102]. Functional polymorphisms in *SLC30A10* impact neurodevelopmental outcomes through manganese dysregulation [39]. Notably, the *SLC30A10* rs1776029 A allele correlates with elevated blood manganese, whereas rs12064812 C is linked to reduced levels [39]. Carriers of manganese‐elevating genotypes demonstrate significant neurodevelopmental deficits, including decreased IQ scores and increased incidence of behavioral disorders.

The ZnT family plays a pivotal role in maintaining zinc and manganese homeostasis across various tissues and is intricately associated with numerous physiological and pathological processes. ZnT1 is essential for zinc efflux and embryonic development, whereas ZnT2 and ZnT4 are critical for mammary gland zinc secretion and lactation. Mutations in the *SLC30A2* gene are a significant cause of TNZD, although no association has been established between *SLC30A4* mutations and TNZD. ZnT3 is linked to neurological functions and susceptibility to schizophrenia, a correlation observed exclusively in females. ZnT5 is vital for cardiac, neuromuscular, and skeletal development, with mutations leading to cardiomyopathy and severe hypotonia. ZnT6 is associated with the severity of RA through zinc‐mediated anti‐inflammatory effects. ZnT7 is crucial for brain and nervous system development, and its mutations are related to JS. ZnT8 plays a key role in insulin storage and secretion and has significant implications in T1DM, T2DM, GDM, and PTDM. ZnT9 is essential for zinc homeostasis in the brain, with mutations causing cerebrorenal syndrome. ZnT10, a crucial manganese transporter, is a key regulator of manganese homeostasis in humans, and its mutations can lead to hypermanganesemia and neurodevelopmental defects. Collectively, the ZnT family underscores the importance of zinc and manganese transport in health and disease, offering potential therapeutic targets for a variety of conditions (Tables 3 and 4).

**Table 3:** The genetic variation of SLC30A and human disease.

ZnT (*SLC30*)	Mutation type	Associated diseases	Clinical symptoms	References
ZnT1 (*SLC30A1*)	Somatic mutations	Primary aldosteronism	Abnormally increased aldosterone synthesis	[67]
ZnT2 (*SLC30A2*)	Loss‐of‐function variants and single nucleotide polymorphisms	Transient neonatal zinc deficiency	Reduced zinc concentration in breast milk, leading to neonatal zinc deficiency symptoms (neuromuscular dysfunction, developmental delay, etc.)	[56, 60, 70]
ZnT3 (*SLC30A3*)	Single nucleotide polymorphisms	Schizophrenia	Increased schizophrenia susceptibility	[59, 73, 74]
ZnT4 (*SLC30A4*)	Gene knockout (mouse model)	Mammary secretion defects and neonatal lethality	Reduced mammary gland weight, milk production, lactose, and zinc content	[8, 9]
ZnT5 (*SLC30A5*)	Biallelic loss‐of‐function variants	Perinatal lethal cardiomyopathy and neonatal hypotonia	Cardiomyopathy leading to fetal demise, neuromuscular dysfunction, developmental delay, and feeding difficulties	[60, 77]
ZnT6 (*SLC30A6*)	Single nucleotide polymorphisms	Rheumatoid arthritis	Dysregulated inflammatory response and lower CDAI scores	[80]
ZnT7 (*SLC30A7*)	Compound heterozygous mutations, missense mutation, and frameshift mutation	Growth retardation, testicular hypoplasia, bone marrow failure, and Joubert syndrome	Developmental disorders, hematopoietic dysfunction, cerebellar and brainstem developmental abnormalities, “molar tooth sign” on MRI, and ataxia	[61, 82]
ZnT8 (*SLC30A8*)	Single nucleotide polymorphisms	Type 2 diabetes mellitus (T2DM), T1DM, GDM, and post‐transplant diabetes mellitus (PTDM)	Abnormal insulin secretion, increased T2DM risk, ZnT8 autoantibodies as early diagnostic markers for T1DM, increased GDM risk, and poor prognosis in PTDM	[12, 62, 84–94]
ZnT9 (*SLC30A9*)	Loss‐of‐function variants	Cerebrorenal syndrome	Early‐onset neurodegeneration, limb ataxia, muscle weakness, and progressive renal dysfunction	[14]
ZnT10 (*SLC30A10*)	Pathogenic variants	Hereditary hypermanganesemia	Manganese deposition in basal ganglia (T1‐weighted hyperintensity on MRI), manifested as hypermanganesemia, polycythemia, hepatic cirrhosis, and neurological symptoms (dystonia, seizures	[39, 63, 97–102]

**Table 4:** Integrative summary of ZnT (SLC30A) and ZIP (SLC39A) transporter gene variants in human disease.

Disease category	Disease	ZnT and ZIP involved	Convergent mechanism	References
Metabolic/endocrine	Type 1 diabetes mellitus (T1DM), Type 2 diabetes mellitus (T2DM), gestational diabetes mellitus (GDM), and post‐transplantation diabetes mellitus (PTDM)	ZnT8 (SLC30A8): rs13266634 and ZIP12 (SLC39A12): Fasting glucose	ZnT8 loads Zn into insulin secretory granules for hexameric insulin crystallization. rs13266634 reduces ZnT8 transport activity, impairing first‐phase insulin secretion (T2DM). ZnT8 serves as a beta‐cell‐specific autoantigen; ZnT8A detected in 60%–80% of T1DM cases. rs13266634 also modulates GDM risk and PTDM outcomes. SLC39A12 rs2497753 and rs9418383 affect fasting blood glucose.	[12, 62, 82–94, 103]
Neurological/psychiatric	Schizophrenia	ZnT3 (SLC30A3): rs6547521, ZIP8 (SLC39A8): rs13107325, and ZIP12 (SLC39A12): Brain expression	ZnT3 impairs presynaptic vesicular Zn loading; ZIP8 disrupts postsynaptic NMDA receptor and immune signaling. Both converge on synaptic Zn dyshomeostasis. ZnT3–Shank3 interaction links to autism spectrum disorder (mouse). ZIP12 protein altered in postmortem schizophrenia brain.	[7, 72, 73, 104–112]
Neurological/psychiatric	Bipolar disorder (BD)	ZIP3 (SLC39A3): rs4806874	SLC39A3 variant associated with BD development. Serum Zn concentration in BD patients is lower than in healthy controls, suggesting Zn homeostasis imbalance in BD pathogenesis.	[22–24]
Neurological/psychiatric	Parkinsonism–dystonia (hypermanganesemia)	ZnT10 (SLC30A10): Biallelic loss‐of‐function (LOF), ZIP14 (SLC39A14): Biallelic LOF, and ZIP8 (SLC39A8): Biallelic LOF	ZnT10: Defective hepatobiliary Mn excretion (Mn efflux) → hypermanganesemia with dystonia, parkinsonism, polycythemia, and cirrhosis. ZIP14: Disrupted hepatic Mn uptake (Mn influx) → hypermanganesemia with dystonia–parkinsonism (predominantly neurological, minimal hepatic involvement). ZIP8: Congenital disorder of Mn metabolism and glycosylation → intellectual disability, cerebellar atrophy (neurodevelopmental, distinct from acquired dystonia).	[40, 41, 52, 63, 97, 100–102, 111, 113–117]
Neurological/psychiatric	Joubert syndrome (JS) and cerebrorenal syndrome (Birk–Landau–Perez)	ZnT7 (SLC30A7): Candidate gene and ZnT9 (SLC30A9): Biallelic LOF	ZnT7 proposed as a candidate gene for JS (ciliopathy with cerebellar/brainstem malformation, molar tooth sign on MRI). ZnT9 mutations disrupt intracellular Zn regulation, causing cerebrorenal syndrome (neurodegeneration, ataxia, and renal dysfunction). Recent case expands phenotype to include chorioretinal degeneration.	[14, 61, 82, 95]
Cardiovascular	Primary aldosteronism, perinatal cardiomyopathy, neonatal hypotonia, and cardiovascular disease/hypertension	ZnT1 (SLC30A1): Somatic deletion, ZnT5 (SLC30A5): Biallelic LOF, and ZIP8 (SLC39A8): rs13107325	ZnT1 somatic mutations → aberrant Na influx → membrane depolarization → Ca^2+^ influx → CYP11B2 upregulation → aldosterone excess. ZnT5 biallelic LOF → cardiac Zn dyshomeostasis → perinatal lethal cardiomyopathy. ZnT5 missense LOF → neonatal hypotonia (neuromuscular dysfunction). ZnT1 variants associated with cardiometabolic diseases (hypertension, dyslipidemia, and coronary heart disease). ZIP8 rs13107325 modulates blood pressure, BMI, and lipid profiles; NT‐proBNP in acute coronary syndrome	[38, 40, 60, 67, 68, 77, 104–111, 113–116, 118–120]
Immune/inflammatory	Rheumatoid arthritis (RA), agammaglobulinemia, and Crohn′s disease (CD)	ZnT6 (SLC30A6): rs430759, ZIP7 (SLC39A7): Biallelic LOF, and ZIP8 (SLC39A8): rs13107325	NF‐*κ*B convergence: ZnT6 A/A genotype → miR‐4760‐3p site abolished → ZnT6 upregulation → enhanced Zn transport → NF‐*κ*B suppression → lower RA severity. ZIP7 mediates ER Zn release essential for B cell development; biallelic LOF → cytosolic Zn depletion → B cell arrest at pre‐B stage → agammaglobulinemia. ZIP8 rs13107325 T allele elevates CD risk, potentially through gut microbiome alterations.	[11, 37, 38, 78–80, 114]
Zn deficiency disorders	Transient neonatal zinc deficiency (TNZD) (infant‐restricted) and acrodermatitis enteropathica (AE) (systemic)	ZnT2 (SLC30A2): Multiple missense and ZIP4 (SLC39A4): Biallelic LOF	ZnT2: Impaired mammary Zn secretion → milk Zn 10%–25% of normal → TNZD in exclusively breastfed infants; mother asymptomatic (other ZnTs maintain maternal homeostasis). ZIP4: Impaired intestinal Zn absorption → systemic AE (dermatitis, diarrhea, and alopecia). Both treatable with Zn supplementation.	[6, 26, 27, 56, 58, 69–71, 121, 122]
Connective tissue/skeletal	Spondylocheirodysplastic Ehlers–Danlos syndrome (SCD‐EDS), high myopia (HM), and adolescent idiopathic scoliosis (AIS)	ZIP5 (SLC39A5): Biallelic LOF, ZIP8 (SLC39A8): rs13107325/rs11097773, and ZIP13 (SLC39A13): Biallelic LOF	BMP/TGF‐beta pathway convergence: ZIP5 mutations impair BMP/TGF‐beta signaling → Smad1 destabilization → impaired scleral extracellular matrix synthesis → HM. Loss of ZIP13 function leads to excessive muscle satellite cell activation and subsequent depletion → SCD‐EDS ZIP8 variants associated with AIS; rs13107325 in Europeans and rs11097773 in Han Chinese.	[28–34, 40, 49–51, 113, 123–126]
Metal overload/toxicology	Hypermanganesemia and Cd/As toxicity	ZnT10 (SLC30A10), ZIP2 (SLC39A2), ZIP8 (SLC39A8), and ZIP14 (SLC39A14)	ZnT10 (Mn efflux) and ZIP14 (Mn uptake) → convergent hypermanganesemia with divergent organ involvement. ZIP2/8/14: Competitive transport of Cd/As → environmental metal susceptibility. ZIP2 rs2234636 TC/CC genotypes increase As‐related bladder Ca risk.	[19, 39, 52–55, 97–102, 119]
Developmental	Growth retardation, testicular hypoplasia, and bone marrow failure	ZnT7 (SLC30A7): Compound het LOF	Compound heterozygous SLC30A7 mutations → growth retardation, testicular hypoplasia, and bone marrow failure. Hormone replacement therapy shows therapeutic benefit.	[82]
Other	Chronic gastritis longevity	ZIP11 (SLC39A11): Intronic variants	SLC39A11 intronic variants (rs17183225, rs17780814, and rs17780820) associated with chronic gastritis; spicy food intake interaction. rs989443 linked to male longevity. ZIP11 identified as a novel Mn transporter; regulates Mn homeostasis and cellular senescence.	[127–129]

## 3. ZIPs (SLC39A)

### 3.1. ZIP1 (*SLC39A1*)

ZIP1 is the first member of the ZIP family to be discovered and widely expressed in a variety of tissues, and its subcellular localization is mainly distributed in organelles and cell membranes (Table 2). It has been found that ZIP1 plays an important role in a variety of cancers, including prostate cancer, glioma, and stomach cancer [16–18]. Compared with normal prostate tissues, prostate cancer tissues have a reduced concentration of zinc and decreased ZIP1 levels [16]. The prostate is the organ with the highest zinc concentration in the body. ZIP1 is the primary ZnT responsible for zinc absorption in prostate tissue and plays a role in suppressing prostate cancer [130]. Golovine et al. found that the overexpression of ZIP1 can reduce the malignant potential of prostate cancer cells by inhibiting NF‐*κ*B activity [131]. In a mouse model, overexpression of ZIP1 was found to suppress growth of prostate tumor xenografts [131]. NF‐*κ*B is an important nuclear transcription factor. The activation of NF‐*κ*B can promote the growth and metastasis of prostate cancer cells by regulating the expression of various tumorigenic cytokines, intercellular adhesion molecule 1, and antiapoptotic proteins [131]. These results suggest that ZIP1 exerts a tumor‐suppressing effect on prostate cancer, possibly related to the inhibition of NF‐*κ*B activity. In contrast to the oncogenic role of ZIP1 in prostate cancer, ZIP1 plays an oncogenic role in glioma and stomach cancer. Wang et al. found that ZIP1 is highly expressed in glioma tissues [17]. The expression of ZIP1 was negatively correlated with the prognosis of glioma patients [17]. Higher ZIP1 expression was observed in patients with worse survival [17]. The results of in vitro experiments demonstrated that ZIP1 promotes the proliferation of glioma cells and inhibits apoptosis [17]. The potential mechanism may be correlated with the upregulation of metalloproteinases MMP‐2 and MMP‐9. MMP‐2 and MMP‐9 are zinc‐dependent enzymes involved in the degradation of extracellular matrix (ECM) proteins, thereby increasing glioma invasion and metastasis [132, 133]. Additionally, ZIP1 may influence the infiltration of immune cells in the glioma microenvironment [17]. Many immune cell activations are positively correlated with ZIP1 expression. These findings suggest that ZIP1 could be a potential therapeutic target for the treatment of gliomas [17]. Yu et al. showed that the overexpression of ZIP1 promotes the proliferation of human gastric cancer (GC) cells in vitro, and the *SLC39A1*‐overexpression nude mouse models have larger tumors, whereas the tumor size in the *SLC39A1*‐knockdown group is significantly reduced [18]. Compared with adjacent stomach tissues, the mRNA and protein levels of ZIP1 are increased in gastric adenocarcinoma tissues and play an oncogenic role by promoting the growth and metastasis of cancer cells [18]. Higher ZIP1 was observed in patients with larger tumor size, advanced tumor stages, and lower survival time [18]. Abnormally high expression of ZIP1 is a risk factor for poor prognosis of stomach cancer, and inhibition of ZIP1 expression may be a new direction for the treatment of patients with GC and the improvement of prognosis. No study has yet found that *SLC39A1* gene variants are associated with human diseases.

### 3.2. ZIP2 (*SLC39A2*)

ZIP2 is an important ZnT protein in the body and is responsible for the uptake of zinc into the cell (Table 2). The *SLC39A2* gene variant has been associated with bladder cancer and carotid artery stenosis [19, 20]. The *SLC39A2* rs2234636 TC and CC genotypes increase the risk of arsenic‐related bladder cancer [19]. ZIP2 is a metal transporter, yet it does not bind to arsenic. Studies have found that arsenic can replace zinc in zinc finger proteins, thus severely disrupting their function [21]. Compared to the homozygous wild‐type TT, heterozygous TC and homozygous CC variants may affect the function of ZIP2 and impact zinc absorption. This can facilitate the replacement of zinc by arsenic in critical metabolic processes, thereby increasing the risk of arsenic‐induced bladder cancer. *SLC39A2* rs2234632 is associated with carotid artery stenosis [20]. Patients with carotid artery stenosis have an increased frequency of the *SLC39A2* rs2234632 GG genotype. The *SLC39A2* rs2234632 GG genotype reduces the zinc uptake function of ZIP2 and leads to zinc deficiency [20]. Zinc deficiency is an important risk factor for carotid artery stenosis.

### 3.3. ZIP3 (*SLC39A3*)

ZIP3 is widely expressed in many tissues and is mainly localized in the cell membrane, responsible for the transport of zinc into the cell (Table 2). *SLC39A3* gene variants are mainly associated with bipolar disorder (BD). Baum et al. found for the first time that *SLC39A3* rs4806874 was closely related to the development of BD [22, 23]. Serum zinc concentration in BD patients is lower than that of normal subjects, suggesting that an imbalance of zinc homeostasis is involved in the pathogenesis of BD [24]. Li et al. found that the *SLC39A3* gene (rs759071, rs10407667, rs4806874, and rs10415622) expression levels were associated with bladder cancer risk. The high expression of *SLC39A3* was associated with lower odds of bladder cancer [25].

### 3.4. ZIP4 (*SLC39A4*)

ZIP4 is highly expressed in the intestinal epithelium, serving as the primary mediator of dietary zinc absorption (Table 2). AE, a rare autosomal recessive disorder, results from defective intestinal zinc absorption, manifesting as characteristic dermatological (perioral, perianal, and acral erythema; vesicles; and crusting lesions) and systemic symptoms (diarrhea and alopecia) [58]. *SLC39A4* mutations represent the principal genetic basis of AE [58]. Extensive genetic analyses have identified diverse *SLC39A4* mutations (deletions, insertions, missense, nonsense, and splice‐site variants) causally associated with AE [26, 27, 122]. ZIP4 can also influence GC development by modulating cuproptosis [134]. YTH N6‐methyladenosine RNA binding protein 1 (YTHDF1) is a key m^6^A reader involved in the development of GC [134]. YTHDF1 recognizes m^6^A modification sites on SLC39A4 mRNA, thereby enhancing its stability and translational efficiency, which leads to ZIP4 overexpression [134]. Overexpressed ZIP4 elevates intracellular zinc concentrations and suppresses cuproptosis through a zinc‐mediated mechanism, thereby enabling GC cells to evade apoptosis and sustain proliferation [134]. The regulation of cuproptosis by ZnTs represents an emerging direction in ZnT research with implications for cancer biology [134].

### 3.5. ZIP5 (*SLC39A5*)

ZIP5 exhibits dynamic expression throughout ocular development, with particularly high abundance in scleral and retinal tissues (Table 2). *SLC39A5* mutations are strongly implicated in high myopia (HM) pathogenesis [28]. HM is clinically defined by a refractive error ≤ −6.00 diopters and/or axial length ≥ 26 mm [29]. HM demonstrates significant heritability, with multiple genetic loci identified through exome sequencing studies, among which *SLC39A5* mutations represent a major genetic contributor. Pioneering work by Guo et al. identified *SLC39A5* mutations (c.141C>G, p. Tyr47 ^∗^ and c.911T>C, p. Met304Thr) segregating with autosomal dominant HM in Chinese pedigrees [28]. Subsequent studies by Jiang et al. revealed a heterozygous missense variant (c.1238G>C, p. Gly413Ala) in a cohort of 298 HM patients [30]. Feng et al. reported three additional heterozygous variants (c.860C>T, p. Pro287Leu; c.956G>C, p. Arg319Thr; and c.250C>T, p. Arg84Trp) in sporadic HM cases [31]. Liu et al. discovered a novel frameshift mutation (c.726dupA, p.Arg243fs ^∗^140) in Tujia Chinese patients with early‐onset HM through whole‐exome sequencing [32]. Zheng et al. characterized two additional frameshift variants (p. Arg1350delC, p. Val451Cysfs76 and c.1023_1024insA, p. Pro342Thrfs41) in sporadic HM cases [33]. Collectively, these findings establish *SLC39A5* as a causative gene for HM. ZIP5 contributes to HM pathogenesis through modulation of BMP/TGF‐*β* signaling. *SLC39A5* mutations compromise ZIP5 function, leading to intracellular zinc dyshomeostasis and subsequent Smad1 protein destabilization [34]. As a critical mediator of BMP/TGF‐*β* signaling, Smad1 dysregulation impairs scleral ECM synthesis, providing a mechanistic basis for HM development [34].

### 3.6. ZIP6 (*SLC39A6*)

ZIP6, also known as LIV‐1, is more widely distributed in vivo, especially expressed in hormone‐regulated tissues (Table 2) [135, 136]. ZIP6 is a downstream target of signal transducer and activator of transcription 3 (STAT3) [137, 138]. The expression of ZIP6 is induced by STAT3. ZIP6 is activated by cleaving on the N‐terminus before locating to the plasma membrane [137, 138]. The activation of ZIP6 promotes zinc influx. This zinc influx leads to the activation of Snail through the inactivation of glycogen synthase kinase 3*β* [137, 138]. Snail is a critical factor in epithelial–mesenchymal transition (EMT), remaining in the cell nucleus and functioning as a transcriptional repressor of E‐cadherin, causing cell rounding and detachment [137, 138]. These results indicate that ZIP6 can affect the progression and metastasis of malignant tumors and the prognosis of patients with malignant tumors by inducing EMT [137, 138]. Wu et al. conducted a GWAS on 1331 patients with esophageal squamous cell carcinoma (ESCC) and found that *SLC39A6* rs1050631 and rs7242481 were correlated with the survival time of ESCC patients [35]. *SLC39A6* rs1050631 CC genotype ESCC patients survived longer than CT or TT genotypes, and rs7242481 GG genotype ESCC patients survived longer than AA or GA genotypes. Gao et al. found that *SLC39A6* rs1050631 also correlated with the prognosis of GC patients after resection [36]. Patients with stomach cancer in the CT or TT genotype had a significantly higher risk of postoperative recurrence and death and a significantly lower overall survival time than patients in the CC genotype [36]. The rs7242481 G>A may obliterate a transcriptional repressor binding site in the 5 ^′^ UTR of SCL39A6, resulting in the up‐expression of *SLC39A6* [35]. The increased expression of ZIP6 leads to larger tumor size and increased metastasis, which is associated with shortened survival time for ESCC patients. Higher expression of ZIP6 in cancerous tissues predicted a poorer prognosis; *SLC39A6* SNPs may improve the prognosis of cancer patients by decreasing the expression of ZIP6 protein and attenuating the invasiveness of cancer cells [35, 36]. ZIP6 overexpression is associated with reduced overall survival and progression‐free survival across multiple tumor types. Hongmin et al. demonstrated that elevated SLC39A6 levels enhance sensitivity to the PI3K*α* inhibitor CH5132799 and further validated in mouse models that CH5132799 effectively suppresses SLC39A6‐mediated tumor growth [139]. These findings advance SLC39A6 from a prognostic indicator to an actionable companion diagnostic biomarker for CH5132799, thereby addressing a critical gap in SLC39A6‐targeted precision oncology [139].

### 3.7. ZIP7 (*SLC39A7*)

ZIP7 demonstrates ubiquitous expression with primary localization to the endoplasmic reticulum and Golgi apparatus, mediating zinc transport from these organelles to the cytoplasm to maintain cytosolic zinc homeostasis (Table 2). Zinc plays a crucial role in regulating immune system function [37]. B cell development is particularly sensitive to zinc concentration fluctuations. *SLC39A7* deletion results in cytoplasmic zinc depletion, arresting B cell development at the pre‐B cell stage and leading to immunodeficiency [37]. Unno et al. first described a novel immunodeficiency syndrome associated with biallelic *SLC39A7* deletions [135]. Affected patients exhibit early‐onset infections, agammaglobulinemia, and B cell deficiency, with severe cases showing cutaneous manifestations, developmental delay, and thrombocytopenia [37]. Erdos et al. identified a novel *SLC39A7* variant (c.1A>G, p. Thr351Ala) associated with recurrent respiratory infections, meningitis, agammaglobulinemia, and B cell lymphopenia [38].

### 3.8. ZIP8 (*SLC39A8*)

ZIP8 functions as a critical metal ion transporter, mediating the transport of multiple divalent cations, including zinc, manganese, iron, selenium, and cadmium (Table 2). Consequently, *SLC39A8* mutations disrupt systemic metal homeostasis, with numerous *SLC39A8* SNPs implicated in metal dysregulation [39, 118–120]. The rs233804 variant modulates zinc and cadmium levels, with A allele carriers exhibiting elevated blood concentrations of both metals compared to noncarriers. The rs13107325 polymorphism influences manganese homeostasis, with T allele carriers demonstrating reduced blood manganese levels [39, 118–120]. *SLC39A8* mutations cause hypomagnesemia, compromising galactosyltransferase activity and leading to congenital disorders of glycosylation manifested as craniofacial abnormalities, seizures, limb shortening, bradykinesia, and hearing impairment [120]. The rs10014145 variant correlates with cadmium metabolism, with G allele carriers showing elevated cadmium levels in blood and urine [119]. Collectively, these findings establish ZIP8 as a key regulator of metal homeostasis, with *SLC39A8* variants primarily exerting their pathogenic effects through metal dysregulation. *SLC39A8* variants have been implicated in diverse pathologies, ranging from neuropsychiatric disorders to musculoskeletal, inflammatory, and cardiometabolic diseases.

*SLC39A8* mutations exhibit strong genetic associations with schizophrenia, a highly heritable neuropsychiatric disorder. Initial evidence from Carrera et al. established the association between *SLC39A8* rs13107325 and schizophrenia in Europeans [104]. This finding was subsequently replicated in a large‐scale case‐control study (37,000 cases; 113,000 controls) by the Psychiatric Genomics Consortium [105]. The rs13107325 variant, located in the coding region, demonstrates extensive pleiotropy, influencing multiple disease phenotypes [106]. The rs13107325 T allele exhibits marked population stratification, with a frequency of ~10% in Europeans but near absence in Asian and African populations [107]. This distribution pattern may reflect positive selection in Europeans, where the T allele confers thermoregulatory advantages and reduces susceptibility to hypertension and metabolic disorders. Thus, the T allele represents an evolutionary adaptation to European environments [107]. However, this adaptive advantage comes with increased schizophrenia risk, likely a pleiotropic trade‐off. The pathogenic mechanisms involve disrupted brain metal homeostasis and neuroimmune dysregulation [108]. The T allele impairs ZIP8 function, reducing zinc influx and glutamate receptor expression, thereby disrupting glutamatergic signaling and contributing to cognitive deficits [108]. Concurrently, ZIP8 dysfunction compromises blood–brain barrier integrity, elevating proinflammatory cytokines and hyperactivating NF‐*κ*B signaling, culminating in neuroimmune dysregulation [108]. These pathophysiological changes represent key mechanisms in schizophrenia pathogenesis [108–110]. Population‐specific genetic architectures underlie schizophrenia risk, with *SLC39A8* emerging as a major risk locus in Europeans. While rs13107325‐T predisposes Europeans to schizophrenia, its absence in Asian and African populations suggests alternative risk factors. In Chinese Uyghurs, a novel *SLC39A8* variant (rs10014145) demonstrated an association with schizophrenia, confirmed by meta‐analysis across Han and Uyghur populations [111]. Thus, rs10014145 represents a population‐specific schizophrenia risk variant, expanding our understanding of *SLC39A8*′s role in schizophrenia [111].

*SLC39A8* mutations demonstrate strong genetic associations with adolescent idiopathic scoliosis (AIS), a complex disorder whose etiology remains incompletely understood, though genetic predisposition plays a significant role. Haller et al. identified *SLC39A8* rs13107325 as a significant risk locus for AIS, with T allele dosage correlating with disease severity [40]. Preclinical studies suggest that this variant impairs manganese transport, potentially contributing to AIS pathogenesis [40]. However, this association was not replicated in Chinese Han populations, where the CC genotype is fixed. Instead, a novel variant (rs11097773) emerged as a population‐specific risk factor in Han Chinese [40]. Reduced ZIP8 expression in paraspinal muscles, correlated with BMI, suggests a potential role in energy metabolism underlying AIS susceptibility in Han Chinese [113].

*SLC39A8* also contributes to Crohn′s disease (CD) susceptibility, a chronic inflammatory bowel disease with strong genetic components [114]. Li et al. demonstrated that the rs13107325 T allele elevates CD risk, potentially through microbiome alterations, as T allele carriers exhibit gut microbiota profiles resembling those of CD patients [114].

*SLC39A8* further influences cardiovascular disease (CVD) risk, a major contributor to global morbidity and mortality. The pleiotropic rs13107325 variant modulates multiple CVD risk factors, including blood pressure, BMI, and lipid profiles [107, 115, 116]. Collectively, these findings establish *SLC39A8* as a key regulator of cardiovascular health. Moreover, rs13107325 impacts outcomes in acute coronary syndromes (ACSs), with T allele carriers showing elevated NT‐proBNP levels predictive of increased mortality [41].

The *SLC39A8* variants further associate with distinct brain MRI phenotypes, suggesting roles in neurodevelopment and function [117]. The c.112G>C (p. Gly38Arg) mutation causes a severe neurodevelopmental disorder characterized by intellectual disability, microcephaly, growth retardation, and hypotonia [117]. Additionally, *SLC39A8* variants predispose to steatohepatitis, HBV‐related hepatocellular carcinoma, and autoimmune disorders [41, 140, 141]. In summary, *SLC39A8* emerges as a pleiotropic gene implicated in diverse pathologies, highlighting the need for mechanistic studies to uncover therapeutic targets for associated genetic disorders.

### 3.9. ZIP9 (*SLC39A9*)

ZIP9, the most compact ZIP family member at ~300 amino acids, represents the sole constituent of the ZIP‐I subfamily (Table 2). Beyond its zinc transport function, ZIP9 serves as a functional membrane androgen receptor. In Sertoli cells, membrane‐localized ZIP9 mediates testosterone signaling, enhancing tight junction protein expression and facilitating blood–testis barrier formation via nonclassical pathways [42]. Thus, ZIP9 contributes to blood–testis barrier homeostasis. ZIP9 demonstrates high expression in melanoma cells, where testosterone‐ZIP9 signaling stimulates proliferation through zinc efflux, MAPK activation, and YAP nuclear translocation [43]. ZIP9 further associates with hepatocellular and epithelial carcinomas, though no pathogenic *SLC39A9* variants have been reported in human diseases [44, 142].

### 3.10. ZIP10 (*SLC39A10*)

ZIP10 belongs to the LIV‐1 subfamily of ZnTs, playing crucial roles in embryogenesis and cutaneous zinc regulation (Table 2). During mammalian oocyte maturation, substantial zinc accumulation, mediated by ZIP10 and ZIP6, is essential for meiotic progression and ovum development [45]. ZIP10–ZIP6 heterodimers further regulate embryonic development, as evidenced by developmental delays and craniofacial, ocular, cardiac, and caudal defects in *SLC39A10*‐silenced zebrafish [46]. ZIP10 also serves as a critical ZnT in hematopoiesis, with *SLC39A10* deletion causing hematopoietic stem cell zinc deficiency, resulting in DNA damage, apoptosis, and cell cycle arrest [47]. Consistent hematopoietic defects are observed in *SLC39A10*‐mutant zebrafish and mice [46, 47]. In skin biology, ZIP10 emerges as a key regulator of epidermal development, being particularly sensitive to zinc status. ZIP10 maintains epidermal homeostasis through modulation of histone acetyltransferase (HAT) activity, with ZIP10 deficiency impairing HAT function, disrupting differentiation programs, and predisposing to dermatological disorders [48]. ZIP10 further contributes to oncogenesis in breast, renal, and thyroid cancers, though no pathogenic *SLC39A10* variants have been associated with human diseases [143–145].

### 3.11. ZIP11 (*SLC39A11*)

ZIP11 is the only member of the gufA subfamily in mammals, and its mRNA is expressed in large quantities in the urinary and digestive systems, so *SLC39A11* gene variants may be associated with urinary and digestive disorders (Table 2) [4]. Wu et al. found that *SLC39A11* gene variants are closely associated with cancers in the urinary system. *SLC39A11* rs8081059 was associated with an increased risk of renal cell carcinoma [64]. Four *SLC39A11* SNPs (rs11871756, rs11077654, rs9913017, and rs4969054) were associated with the risk of bladder cancer. rs11871756 was associated with an increased risk of bladder cancer. rs11077654, rs9913017, and rs4969054 are associated with a decreased risk of bladder cancer. In addition, the *SLC39A11* gene variants have been associated with the development of chronic gastritis in the Korean population [127]. The rs17183225 (C/T), rs17780814 (A/C), and rs17780820 (A/G) are closely located in the intron region of the *SLC39A11* and show linkage disequilibrium. The prevalence of chronic gastritis in the population with TCG and TCA genotypes is higher than that with CAA genotypes, suggesting that people with the TCG + TCA genotype should increase zinc intake to prevent chronic gastritis [127]. Zeng et al. found that the *SLC39A11* rs989443 polymorphism is linked to longevity in males [128]. The study reveals that the *SLC39A11* gene exerts antiaging effects by regulating manganese homeostasis [129]. Reduced expression of *SLC39A11* leads to manganese accumulation in the serum, increasing DNA damage and reactive oxygen species signaling, thereby driving cellular senescence [129]. In zebrafish, *SLC39A11* mutations result in progeroid phenotypes, such as shortened lifespan, muscle atrophy, and impaired regeneration, with males being more significantly affected [129]. Experiments demonstrate that knocking down *SLC39A11* or exposure to high manganese environments promotes senescence in human fibroblasts, confirming that *SLC39A11* delays aging by modulating manganese levels [129].

### 3.12. ZIP12 (*SLC39A12*)

ZIP12 demonstrates high expression in the brain, fulfilling neuronal zinc demands during neurodevelopment (Table 2). Consequently, *SLC39A12* variants potentially influence brain structure and function [146]. Elliott et al. first identified *SLC39A12* SNPs correlating with brain MRI phenotypes [147]. Strong et al. further demonstrated *SLC39A12* SNP associations with swMRI signals in the caudate nucleus, putamen, and globus pallidus [148]. These variants modulate ZIP12‐mediated zinc transport, impacting human brain structure and function. ZIP12 is highly expressed in the cerebral cortex of patients with schizophrenia, which may represent an important contributor to the disruption of central nervous system zinc homeostasis—a disturbance that is considered to be part of the pathophysiological mechanism underlying schizophrenia [112]. Moreover, intronic variants rs2497753 and rs9418383 influence fasting glucose levels, a key risk factor for T2DM [103].

### 3.13. ZIP13 (*SLC39A13*)

ZIP13, critical for skeletal, dental, and connective tissue development, has *SLC39A13* mutations implicated in SCD‐EDS (Table 2) [49]. EDS encompasses a spectrum of inherited connective tissue disorders [49]. Clinically, EDS is classified into six subtypes, with SCD‐EDS caused by *SLC39A13* mutations [123]. Multiple case reports document *SLC39A13*‐associated SCD‐EDS, characterized by classic EDS features and skeletal abnormalities [49–51, 57]. Characteristic EDS features include hyperelastic skin, joint hypermobility, muscle atrophy, arachnodactyly, proptosis, and blue sclera [124]. Skeletal manifestations comprise short stature, hypotonia, limb contractures, osteopenia, and developmental delays [57]. ZIP13 is a marker of muscle satellite cells (MuSCs) and plays a critical regulatory role in the transition of MuSCs from quiescence to activation [125]. Loss of ZIP13 function leads to excessive MuSC activation and subsequent depletion, thereby impairing muscle regenerative capacity [125]. This finding provides a cellular‐level explanation for the pathophysiological mechanisms underlying muscle atrophy and weakness observed in patients with SCD‐EDS harboring *SLC39A13* mutations [125]. *SLC39A13* mutations trigger ubiquitin–proteasome‐mediated degradation of valine‐containing proteins, reducing functional protein levels and disrupting zinc homeostasis, ultimately causing ECD‐EDS [126].

### 3.14. ZIP14 (*SLC39A14*)

ZIP14, a multifunctional metal transporter, mediates zinc, manganese, iron, and cadmium transport (Table 2). *SLC39A14* variants impair ZIP14 function, disrupting systemic metal homeostasis. The *SLC39A14* SNPs modulate erythrocyte cadmium levels, with rs4872479‐GT/TT and rs870215‐AA/AG genotypes associated with elevated cadmium levels [119]. Moreover, as a primary manganese transporter, *SLC39A14* mutations cause manganese dyshomeostasis [52]. Tuschl et al. identified *SLC39A14* mutations causing hypermanganesemia, parkinsonism, and dystonia [52]. Affected individuals exhibit developmental delay, early‐onset dystonia, and brainstem dysfunction, progressing to generalized dystonia, contractures, and scoliosis [52]. Parkinsonian features (hypomimia, tremor, and bradykinesia) may also emerge [52]. Characteristic findings include elevated blood manganese and basal ganglia accumulation on MRI [52]. Subsequent case reports have confirmed *SLC39A14*‐associated manganese accumulation [53–55]. Unlike *SLC30A10* mutations, *SLC39A14* variants typically lack erythrocytosis and hepatic involvement.

The ZIP family plays a pivotal role in maintaining zinc and manganese homeostasis, with each member exhibiting tissue‐specific expression and functional diversity. ZIP1, ZIP2, ZIP6, and ZIP11 are primarily associated with cancer. ZIP3 is mainly linked to BD and bladder cancer development. ZIP4 serves as the primary mediator of intestinal zinc absorption, and mutations in *SLC39A4* constitute the genetic basis of AE. ZIP5 influences HM by modulating the BMP/TGF‐*β* signaling pathway. ZIP7 is critical for immune function, and deficiency in SLC39A7 leads to severe immunodeficiency syndromes. ZIP8 is essential for metal regulation and is associated with schizophrenia, AIS, CD, and CVD. ZIP9 plays a role in reproductive health, whereas ZIP10 is involved in embryogenesis. ZIP12 is crucial for brain and nervous system development. ZIP13 is vital for connective tissue integrity, and its variants are linked to SCD‐EDS. ZIP14, a multifunctional metal transporter, is implicated in manganese dysregulation and neurological disorders. Collectively, the ZIP family underscores the importance of metal homeostasis, particularly zinc and manganese, in human health and disease (Tables 5 and 4).

**Table 5:** The genetic variation of SLC39A and human disease.

ZIP (*SLC39A*)	Mutation type	Associated diseases	Clinical symptoms	References
ZIP1 (*SLC39A1*)	No identified pathogenic variants	Prostate cancer, glioma, and stomach cancer	ZIP1 overexpression inhibits prostate cancer and promotes glioma/stomach cancer cell proliferation	[16–18, 131]
ZIP2 (*SLC39A2*)	Single nucleotide polymorphisms	Arsenic‐related bladder cancer and carotid artery stenosis	Altered zinc absorption facilitates arsenic toxicity; reduced zinc uptake linked to carotid artery stenosis	[20, 21]
ZIP3 (*SLC39A3*)	Single nucleotide polymorphisms	Bipolar disorder (BD) and bladder cancer	Lower serum zinc in BD; high SLC39A3 expression associated with reduced bladder cancer risk	[22–25]
ZIP4 (*SLC39A4*)	Deletions, insertions, missense, nonsense, and splice‐site variants	Acrodermatitis enteropathica (AE)	Perioral, perianal, and acral erythema; vesicles; and crusting lesions, diarrhea, and alopecia	[26, 27, 58, 122]
ZIP5 (*SLC39A5*)	Nonsense mutation, missense mutation, and frameshift mutation	High myopia (HM)	Refractive error ≤ −6.00 D, axial length ≥ 26 mm, and scleral ECM dysregulation	[28–34]
ZIP6 (*SLC39A6*)	Single nucleotide polymorphisms	Esophageal squamous cell carcinoma (ESCC) and gastric cancer	Genotypes associated with longer survival, and ZIP6 overexpression promotes tumor metastasis	[35, 36]
ZIP7 (*SLC39A7*)	Missense mutation and biallelic deletions	Immunodeficiency syndrome	Early‐onset infections, agammaglobulinemia, B cell deficiency, and thrombocytopenia	[37, 38]
ZIP8 (*SLC39A8*)	Single nucleotide polymorphisms	Idiopathic scoliosis (AIS) and Crohn′s disease (CD)	Disrupted brain metal homeostasis, impaired manganese transport, altered gut microbiota, and pleiotropic effects on CVD risk factors	[39–41, 104–111, 113–120]
ZIP9 (*SLC39A9*)	No identified pathogenic variants	Melanoma and hepatocellular and epithelial carcinomas	ZIP9 demonstrates high expression in melanoma cells and associates with hepatocellular and epithelial carcinomas	[43, 44, 142]
ZIP10 (*SLC39A10*)	No identified pathogenic variants	Embryonic development disorder and hematopoietic arrest	SLC30A10 gene mutations cause developmental delays and body defects, along with hematopoietic defects in zebrafish	[46, 47]
ZIP11 (*SLC39A11*)	Single nucleotide polymorphisms	Renal cell carcinoma, bladder cancer, and chronic gastritis	Increase the risk of renal cell carcinoma, bladder cancer, and gastritis	[4, 64, 127–129]
ZIP12 (*SLC39A12*)	Single nucleotide polymorphisms	Brain structure and function	Variants modulate ZIP12‐mediated zinc transport, impacting human brain structure and function	[147, 148]
ZIP13 (*SLC39A13*)	Various loss‐of‐function mutations	Spondylocheirodysplastic Ehlers–Danlos syndrome (SCD‐EDS)	Hyperelastic skin, joint hypermobility, arachnodactyly, blue sclera, and skeletal deformities	[49–51, 57, 123–126]
ZIP14 (*SLC39A14*)	Single nucleotide polymorphisms and loss‐of‐function variants	Hypermanganesemia, parkinsonism, and dystonia	Developmental delay, early‐onset dystonia, brainstem dysfunction, and basal ganglia manganese deposition	[52–55]

## 4. Comparative and Integrative Roles of ZnT and ZIP Transporters in Human Diseases

### 4.1. Convergent Disease Phenotypes and Metal Cross‐Talk

A key insight emerging from the systematic analysis of the relationship between ZnT gene variants and human disease is that mutations in transporters with opposing functions can produce convergent disease phenotypes, indicating their involvement in shared pathophysiological mechanisms. Schizophrenia exemplifies this principle. ZnT3 reduces cytosolic zinc concentrations by loading Zn into presynaptic vesicles, whereas ZIP8 elevates cytosolic zinc levels by importing zinc into cells. Multiple independent lines of evidence have established that both transporters are genetically associated with schizophrenia [59, 104, 105]. In European populations, SLC30A3 polymorphisms (rs6547521, rs11126936, rs2083363, and rs11126931) exhibit a female‐specific association with schizophrenia [59, 73], whereas the SLC39A8 rs13107325 (p.Ala391Thr) missense variant has been identified as a schizophrenia risk locus in large‐scale GWAS [104, 105]. Despite their opposing directions of zinc transport, both variants ultimately disrupt synaptic zinc signaling. ZnT3 dysfunction impairs presynaptic vesicular zinc loading, whereas ZIP8 deficiency in postsynaptic cells disrupts zinc‐dependent N‐methyl‐D‐aspartate receptors and immune signaling [109]. Both mechanisms converge on synaptic zinc dyshomeostasis, representing a shared vulnerability factor in the pathophysiology of psychosis (Table 4).

Disorders of manganese homeostasis provide an even more compelling example of how mutations in genes from different ZnT families can give rise to similar disease phenotypes. ZnT10 (SLC30A10), a ZnT that also functions as the primary cellular manganese efflux transporter, is associated with mutations causing hypermanganesemia accompanied by dystonia, parkinsonism, polycythemia, and chronic liver disease [63, 98]. Similarly, ZIP14 (SLC39A14), a zinc/manganese influx transporter, is linked to mutations resulting in childhood‐onset hypermanganesemia with parkinsonism–dystonia syndrome [126]. Both disorders are characterized by elevated blood manganese levels and exhibit characteristic T1‐weighted hyperintensity in the basal ganglia on brain MRI [99, 126]. However, their clinical presentations differ markedly: SLC30A10 mutations are accompanied by polycythemia and hepatic cirrhosis in addition to hypermanganesemia [98], whereas SLC39A14 mutations manifest predominantly as neurological disease with minimal hepatic or hematological involvement. These phenotypic differences reflect the distinct expression profiles of ZnT10 and ZIP14. ZnT10 is broadly expressed across tissues and plays roles in hepatobiliary manganese excretion and erythropoiesis, whereas ZIP14 is predominantly expressed in the liver, with additional expression in the brain and intestine. The two transporters mediate opposing directions of manganese flux across cellular membranes: ZnT10 facilitates manganese efflux from cells, whereas ZIP14 mediates manganese influx [15, 149]. The finding that both SLC30A10 and SLC39A14 mutations lead to manganese accumulation in the basal ganglia demonstrates that cerebral manganese homeostasis depends on a balanced, bidirectional transport process mediated jointly by ZnT and ZIP family members. Moreover, SLC39A8 mutations cause a congenital disorder of manganese metabolism characterized by intellectual disability and cerebellar atrophy [117, 120]—a neurodevelopmental rather than neurodegenerative phenotype—further underscoring the distinct developmental roles of ZIP8, ZIP14, and ZnT10 in manganese homeostasis (Table 4).

In addition to manganese, ZIP transporters also mediate the cellular uptake of cadmium, a toxic heavy metal. The SLC39A8 rs13107325 variant influences blood cadmium levels and intracellular cadmium toxicity [106], whereas SLC39A14 polymorphisms modulate cadmium accumulation in erythrocytes [119]. Zinc and cadmium compete for ZIP‐mediated transmembrane transport, and cadmium exposure can disrupt zinc homeostasis by competitively inhibiting ZIP8‐ and ZIP14‐mediated zinc uptake [106]. Furthermore, SLC39A2 SNPs have been identified as modifiers of arsenic‐associated bladder cancer risk: the SLC39A2 rs2234636 TC and CC genotypes increase the risk of arsenic‐related bladder cancer, further expanding the role of ZIP transporters in susceptibility to metal toxicity [19]. This metal–metal interplay extends the pathophysiological relevance of ZnT/ZIP gene variants beyond zinc homeostasis per se, positioning these transporters as critical determinants of individual susceptibility to environmental metal exposure.

### 4.2. Convergent Signaling Pathways in ZnT/ZIP‐Related Diseases

Despite the functional diversity of ZnT and ZIP family members, a limited number of key intracellular signaling pathways emerge as critical nodes through which transporter dysfunction translates into the pathophysiological mechanisms underlying disease processes across multiple organ systems.

The NF‐*κ*B pathway, a central regulator of inflammation and immune responses, represents the most prominent point of convergence. In RA, SLC30A6 modulates NF‐*κ*B activation: The SLC30A6 rs430759 A/A genotype upregulates ZnT6 expression by abolishing the miR‐4760‐3p binding site in the 3 ^′^ UTR, and elevated ZnT6 expression enhances zinc transport while suppressing NF‐*κ*B‐mediated proinflammatory cytokine production, thereby correlating with lower disease severity [80]. In prostate cancer, ZIP1 suppresses the malignant potential of prostate cancer cells by increasing cytosolic zinc concentrations. Elevated cytosolic zinc interferes with I*κ*B kinase phosphorylation, thereby inhibiting NF‐*κ*B activity and reducing the expression of proinvasive genes, including MMP‐2 and MMP‐9 [131–133]. ZIP8 further participates in immune signaling through its association with CD and its essential role in myeloid innate immune cell function during bacterial pneumonia [114, 150]. Despite their opposing directions of zinc transport—ZnT6 mediates efflux, whereas ZIP1 and ZIP8 mediate influx—all three converge on the NF‐*κ*B pathway, indicating that it is the absolute cytosolic zinc concentration within specific subcellular compartments, rather than the direction of transport per se, that determines pathway activity.

The EMT pathway represents another major point of convergence. ZIP6 promotes EMT through STAT3–Snail signaling in breast cancer, pancreatic cancer, and esophageal cancer [30, 31, 33]. ZIP10 reinforces EMT via heterodimerization with ZIP6 [46] and is associated with tumor aggressiveness in breast cancer, renal cell carcinoma, and thyroid cancer [143–145]. In contrast, ZnT2 (SLC30A2) maintains epithelial integrity by limiting cytosolic zinc availability; SLC30A2 variants (rs35235055 and rs35623192) impair zinc sequestration, thereby increasing susceptibility to epithelial dysfunction [69, 121]. This ZnT2/ZIP6/ZIP10 axis constitutes a zinc‐dependent EMT rheostat, and evidence indicates that zinc‐dependent coactivation of the ZEB1 and YAP1 transcriptional regulators drives EMT plasticity and metastatic capacity in pancreatic cancer, directly linking ZnT activity to the core EMT transcriptional regulatory network [151].

## 5. Summary and Outlook

ZnT proteins play crucial roles in zinc ion transport and homeostasis while also mediating the transport of various other metal ions. Emerging genetic evidence has established significant associations between ZnT genes and human diseases, highlighting their potential as novel therapeutic targets. However, the molecular mechanisms underlying ZnT‐related diseases remain incompletely characterized. Elucidating these mechanisms represents a key focus for future investigations. Deciphering ZnT–disease relationships will provide the foundation for identifying therapeutic targets and developing innovative treatment strategies.

## Author Contributions

Xiaohong Bai and Shusong Wang formulated the research question. Bo Sun and Jing Ma performed the literature search and wrote the initial version of the manuscript. Xiaohong Bai and Shusong Wang contributed to the revisions of the manuscript.

## Funding

This study was funded by the Tianjin Medical University General Hospital (22TSC010), Hebei Provincial Project for Introducing Foreign Intelligence and the S&T Program of Hebei (10.13039/501100008238) (246Z7705G).

## Disclosure

All authors read and approved the final manuscript. All authors have agreed to publication.

## Ethics Statement

The authors have nothing to report.

## Conflicts of Interest

The authors declare no conflicts of interest.

## Data Availability

No datasets were generated or analyzed during the current study.
